# A retrospective clinical study of dolutegravir- versus efavirenz-based regimen in treatment-naïve patients with advanced HIV infection in Nanjing, China

**DOI:** 10.3389/fimmu.2022.1033098

**Published:** 2023-01-09

**Authors:** Mingli Zhong, Mengqing Li, Mingxue Qi, Yifan Su, Nawei Yu, Ru Lv, Zi Ye, Xiang Zhang, Xinglian Xu, Cong Cheng, Chen Chen, Hongxia Wei

**Affiliations:** ^1^ Department of Infectious Disease, The Second Hospital of Nanjing, School of Public Health, Nanjing Medical University, Nanjing, China; ^2^ Department of Infectious Disease, The Second Hospital of Nanjing, Nanjing University of Chinese Medicine, Nanjing, China

**Keywords:** efavirenz, dolutegravir, advanced HIV infection, antiretroviral therapy, immune recovery, IRIS - immune reconstitution inflammatory syndrome

## Abstract

Currently, there are limited data related to the efficacy and safety of ART regimens, as well as factors influencing immune recovery in antiretroviral therapy (ART)-naïve patients with advanced HIV infection, especially in China. We designed a single-center, retrospective cohort study from March 1, 2019, to May 31, 2022, at The Second Hospital of Nanjing, China. ART-naïve adults with advanced HIV infection (CD4+ T-cell count < 200 cells/μL) who met the study criteria were included. The plasma viral load (VL), CD4+ T-cell count, CD4/CD8 ratio, treatment discontinuation, and immune reconstitution inflammatory syndrome (IRIS) events were collected to compare the efficacy and safety of the dolutegravir (DTG) and the efavirenz (EFV) regimens. Factors of immune recovery were analyzed using the Cox regression model. Study enrolled 285 ART-naïve adults with advanced HIV-1 infection, of which 95 (33.3%) started regimens including DTG and 190 (66.7%) were treated with EFV. After ART initiation, the proportion of patients with HIV-1 RNA < 50 copies/mL was higher (22.5% versus 6.5%, *P* < 0.001) in those on DTG-based regimens at month 1, but no significant difference at other follow-up points. Compared to the baseline, the median CD4+ T-cell count and CD4/CD8 ratio increased significantly during follow-up both in the EFV and the DTG groups. However, the CD4+ T-cell count increased greater in patients on DTG-based regimens at months 6, 12, 24, and 36 (*P* < 0.05). A total of 52 (18.2%) patients discontinued treatment, with no significant difference between ART regimens in treatment discontinuation rates. Only 7 patients reported IRIS, without significant difference between ART regimens (*P*=0.224). Overall, 34.0% (97/285) achieved a CD4+ T-cell count ≥ 350 cells/μL during follow-up. Age (*P* < 0.001), baseline CD4+ T-cell count (*P* < 0.001), baseline VL (*P* < 0.001) and ART regimens (*P* = 0.019) were associated with the CD4+ T-cell count ≥ 350 cells/μL after adjusting for potential confounders. Among ART-naïve adults with advanced HIV infection, it appeared that DTG-based regimens were better options for initial therapy compared to regimens including EFV; in addition, ART regimens, age, baseline VL and CD4+ T-cell count were associated with immune recovery.

## Introduction

The advent of highly active antiretroviral therapy (HAART) has transformed acquired immunodeficiency syndrome (AIDS) into a manageable and chronic disease (1). Treatment efficacy continues to improve with the update of antiviral treatment drugs, but patients with advanced HIV infection before antiretroviral therapy (ART) initiation are of concern. As defined by The European Late Presenter Consensus working group, patients with advanced HIV infection are those who CD4+ T-cell count < 200 cells/μL or with an AIDS-defining event (2).

Advanced HIV infection is a common phenomenon among ART-naïve people living with HIV (PLWH). In high-income countries, the proportion of PLWH with CD4 less than 200 was already more than 20% (3), which should be even larger in middle and low-income areas. In China, the proportion of late ART initiation had shown a decreasing trend in recent years, from 80% in 2008 to 45% in 2014 (4), and to 29% in 2016 (5). Currently, there are limited data related to the selection, efficacy, and safety of ART regimens specifically for patients with advanced HIV infection. Three clinical trials (6–8) were designed to compare virological efficacy in ART-naïve PLWH with CD4 count < 200 cells/uL, such as the PRADAR study (7) which ART regimens contained abacavir/lamivudine (ABC/3TC) plus darunavir/ritonavir (DRV/r) or raltegravir (RAL), and real-world observational studies (9, 10) mainly conducted in high-income countries. The common regimens used in PLWH were 3-drug regimens (3DRs) including efavirenz (EFV) or dolutegravir (DTG) plus 2 nucleoside reverse transcriptase inhibitors (NRTIs) between 2019 and 2021 in China, while data on the clinical efficacy and safety of common options in advanced ART-naïve PLWH have not been reported.

All PLWH with detectable plasma virus were recommended to receive ART by Recommendations of the International Antiviral Society–USA Panel recommended in 2016 (11). Virological suppression is easily achieved with long-term regular ART in both early and advanced PLWH, but full recovery of the immune system is difficult to achieve, especially in patients with advanced HIV infection (12). Studies showed low CD4+ T-cell count was strongly associated with disease prognosis (13), making HIV patients more susceptible to virological failure (14–16), opportunistic infections (17–19), tumors (20), and even death (13, 21). Therefore, it’s important for advanced ART-naïve PLWH to identify the factors associated with immune recovery and to take proactive measures, especially for PLWH with low CD4+ T-cell count.

The Second Hospital of Nanjing is a tertiary hospital for infectious diseases and a specific admission site for PLWH, admitting and following up the vast majority of PLWH in Nanjing, China. And it is the provincial treatment demonstration site, responsible for the technical guidance of ART for PLWH in Jiangsu Province. The aims of our study were (i) to compare virologic efficacy, immunologic response, and safety with 3DRs including EFV and DTG in ART-naïve PLWH with CD4+ T-cell counts <200 cells/μL and (ii) to explore factors associated with immune recovery in ART-naïve advanced PLWH in Nanjing, China.

## Methods

### Study design and population

This was a single-center, retrospective cohort study conducted from March 1, 2019, to May 31, 2022, at The Second Hospital of Nanjing, China. The inclusion criteria of this study were as follows: ART-naïve PLWH aged at least 18 years; advanced HIV infection, defined as a CD4+ T-cell count < 200 cells/μL at the time of ART initiation between March 1, 2019, and May 31, 2021; initiating 3DRs including EFV (600mg or 400mg) or DTG plus 2 NRTIs and the later included 3TC plus ABC or zidovudine or tenofovir disoproxil fumarate (TDF) or tenofovir alafenamide (TAF); not allergic to any of the drugs who chose; and without baseline resistance to EFV if EFV was selected. PLWH initiated ART with alanine transaminase (ALT) or aspartate transaminase (AST) greater than 5 times the upper limit of normal, estimated glomerular filtration rate (eGFR) less than 50 mL/min/1.73m^2^, without baseline data of HIV-RNA or CD4+ T-cell count, with baseline resistance to EFV if EFV was selected, or allergy to any drug were excluded. Before ART initiation, we performed Sanger sequencing on pre-treatment peripheral blood samples from ART-naive PLWH. The sequences obtained were compared online with the Stanford Drug Resistance Database (http://HIVDB.stanford.edu/) for resistance mutation sites and types, and the resistance levels were classified into five grades: sensitive, potentially low resistance, low resistance, moderate resistance, and high resistance based on the resistance score. Our study defined the latter four grades mentioned above as drug resistance. However, baseline drug resistance was not tested for DTG. The study was approved by the Ethics Committee of the Second Hospital of Nanjing (2019-LS-ky007). All patients provided a signed written informed consent form for the use of their data.

### Variables, definitions and outcomes

Baseline was defined as the date of ART initiation, and baseline demographic data, as well as clinical characteristics [including age, gender, route of transmission, body mass index (BMI), hepatitis B virus (HBV) or hepatitis C virus (HCV) infection, the time between diagnosis and ART initiation, CD4+ T-cell count, CD4/CD8 ratio, HIV-1 viral load (VL), and co-infection with opportunistic infections] were collected from patients who met the inclusion criteria. After ART initiation, data of CD4+ T-cell count, CD4/CD8 ratio, VL, and ART regimens were collected from patients’ electronic medical record systems or paper medical record files at any follow-up point. Follow-up ended at modification of ART regimens (i.e., substitution, addition, or discontinuation of any drug), discontinuation of ART > 30 days, loss of follow-up, death, or study end (i.e., May 31, 2022), whichever above event came first.

Efficacy evaluations included virological suppression and immunologic response. Virological suppression was defined as plasma HIV-1 VL < 50 copies/mL and evaluated at months 1, 3, 6, 12, 24, and 36 after initiation of treatment. Median CD4+ T-cell count and CD4/CD8 ratio were used for evaluating immunologic response and median change from baseline evaluated after 3, 6, 12, 24, and 36 months. Safety evaluations included the frequency of treatment discontinuation and immune reconstitution inflammatory syndrome (IRIS) events. Treatment discontinuation was defined as either modification of the initial ART regimens for any reason (i.e., substitution, addition, or discontinuation of any drug) or discontinuation of ART > 30 days without any ART prescription or death; and the rate of treatment discontinuation was compared between the EFV and the DTG groups within 12 months of treatment and after 12 months, respectively. IRIS was defined as various inflammatory symptoms in PLWH during the recovery of immune function after ART initiation, mainly manifested as fever, the appearance of latent infection, or the aggravation or worsening of the original infection (22). IRIS events were checked in the patient’s inpatient and outpatient electronic medical record system and paper medical record file. Immune recovery was defined as achieving a CD4+ T-cell count ≥ 350 cells/μL for the first time, and multivariate analysis was performed to identify factors associated with immune recovery.

### Statistical analysis

Categorical variables were described by n (%) and compared between groups by Chi-Square Test or Fisher’s exact test. Continuous variables were tested for normality. Normal data were described by mean (standard deviation) and compared between groups by t-test or one-way ANOVA, median [interquartile range (IQR)] and rank sum test were used to describe and compare non-normal data. CD4+ T-cell count and CD4/CD8 ratio at each follow-up point after ART initiation were compared with baseline using the Wilcoxon Matched-pairs Signed-Ranks Test for each of the two regimens. Comparisons of CD4+ T-cell count and CD4/CD8 ratio between the two regimens were performed with the Mann–Whitney U test, due to non-normal distribution. Kaplan-Meier curves and log-rank tests were used to analyze the cumulative probability of achieving immune recovery after ART initiation. In addition, the effects of baseline variables on immune recovery were examined using the Cox regression model. The Schoenfeld residual test was used to verify the assumption of proportional hazards (PH) of each variable and the covariance inflation factor (VIF) was used for covariance diagnosis, with VIF > 10 considered to have covariance. The results showed that CD4+ T-cell count (per 50 cells/μL increase) did not satisfy the PH hypothesis based on a *P* value threshold of 0.05 and the VIF of all variables ranged from 1.041 to 1.801, which can be considered as no covariance among the included variables. Based on the above results, the univariate analysis contained all baseline variables and a time-dependent covariate [CD4+ T-cell count*LN (T_)], and the likelihood ratio forward method was used for multivariate regression analysis incorporating covariates with *P* < 0.2 in univariate analysis. Most patients were treated with DTG or EFV plus 3TC and TDF, so drug classes of NRTIs were not included in the analysis. Furthermore, some patients were taking low-dose EFV (400mg), which we compared with DTG for additional results. All statistical analysis was performed using SPSS Statistics for Windows, version 24.0 (IBM Corp, Armonk, NY, USA) and GraphPad prism 8.0.1.

## Results

### Study population

**Figure 1:**
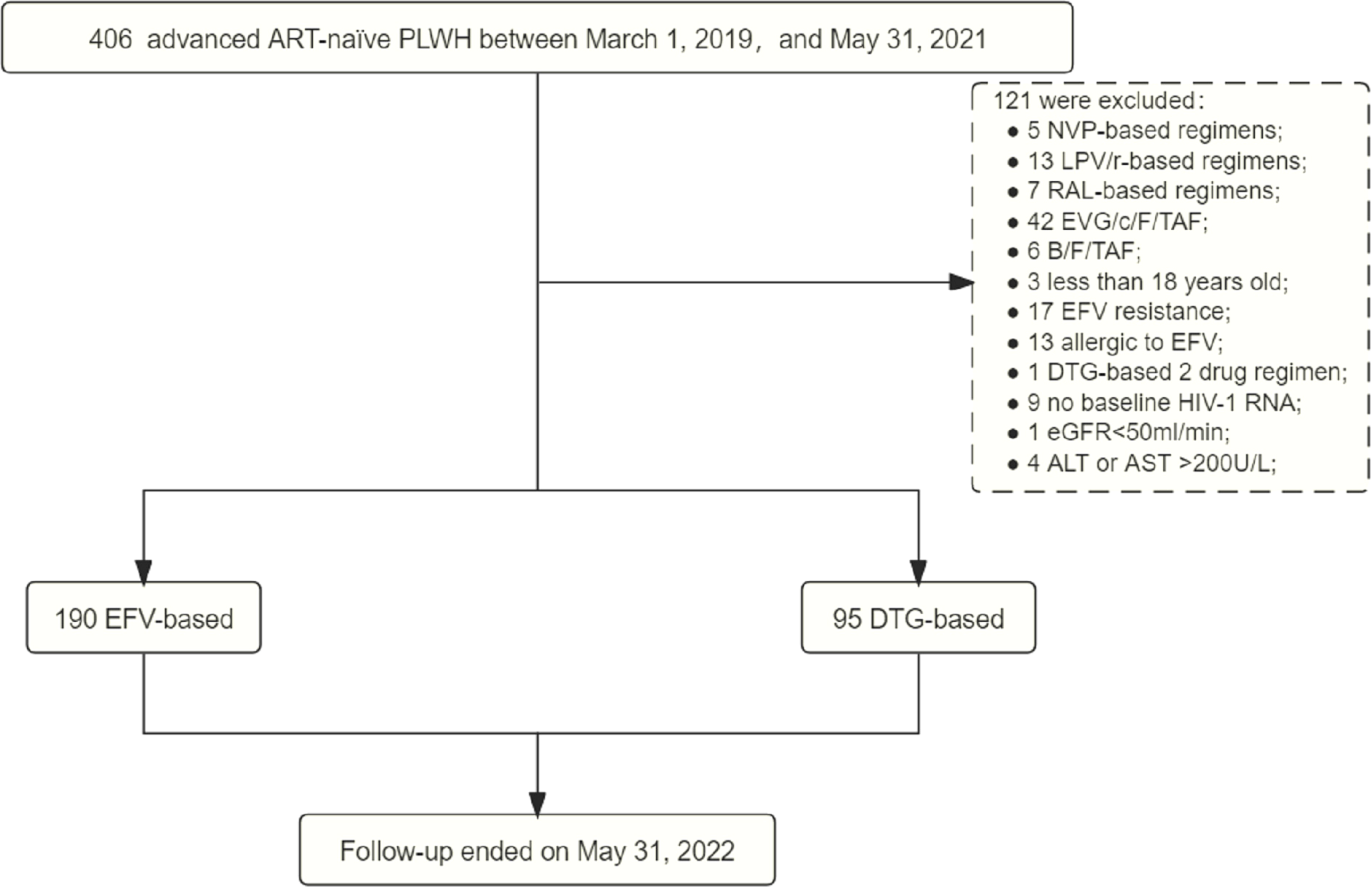
Study process. ART, antiretroviral therapy; PLWH, people living with HIV; NVP, nevirapine; LPV/r, lopinave/litonawe; RAL, raltegravir; EVG/c/F/TAF, elvitegravir/cobicistat/emtricitabine/tenofovir alafenamide; B/F/TAF, bictegravir/emtricitabine/tenofovir alafenamide; EFV, efavirenz; DTG, dolutegravir; eGFR, estimated glomerular filtration rate; ALT, alanine transaminase; AST, aspartate transaminase.

Between March 1, 2019, and May 31, 2021, 406 (32.04%, 406/1267) were advanced ART-naïve PLWH with CD4+ T-cell count < 200 cells/μL, of which 285 met the inclusion criteria, including 190 on EFV-based 3DRs and 95 on DTG-based 3DRs (**Figure 1**). Of the 190 patients on EFV-based 3DRs, 24.2% (46/190) of patients with 400mg EFV and 75.8% (144/190) with 600mg EFV; 33 were not tested for baseline resistance and the rest were not resistant to EFV at baseline.

Overall, median age was 37.0 years (IQR 28.0-54.0), 254 (89.1%) males, 170 (59.6%) homosexuals, median BMI 21.6 Kg/M^2^ (IQR 19.6-23.9). 33 (11.6%) were HBV coinfected, 14 (4.9%) HCV coinfected. Median time between diagnosis and initiation of ART was 26.0 days (IQR 13.0-68.0), median CD4+ T-cell count 84.0 cells/μL (IQR 25.5-145.0) and CD4+ T-cell count were ≤ 50 cells/μL in 100 (35.1%). Median CD4/CD8 ratio was 0.12 (IQR 0.05-0.18) and CD4/CD8 ratio ≤ 0.1 in 134 (47.0%). Median HIV-1 viral load (VL) was 5.0 Log_10_ copies/mL (IQR 4.6-5.4) and VL ≥ 100,000 copies/mL in 146 (51.2%). 144 (50.5%) developed opportunistic infections at baseline, **Table 1**. Demographic characteristics were similar in the EFV and the DTG groups. However, baseline CD4+ T-cell count [56.0 cells/μL (IQR 18.0-117.0) versus 103.0 cells/μL (IQR 36.8-155.5)] and CD4/CD8 ratio [0.09 (IQR 0.04-0.14) versus 0.13 (IQR 0.06-0.19)] were lower, and VL [5.2 Log_10_ copies/mL (IQR 4.8-5.6) versus 5.0 Log_10_ copies/mL (IQR 4.6-5.3)] was higher in those on DTG-based regimens. For patients with low dose EFV (400mg), whose median BMI (*P* = 0.016), rates of high VL (VL ≥ 100,000 copies/mL, *P*=0.014) and opportunistic infections (*P* < 0.001) were lower than with DTG, while median CD4+ T-cell count (*P* = 0.002) and CD4/CD8 ratio (*P* < 0.001) were higher (**Supplementary Table 1**).

**Table 1:** Baseline demographic and clinical characteristics.

	Total (n=285)	EFV (n=190)	DTG (n=95)
Age, median (IQR), y	37.0 (28.0-54.0)	40.0 (28.0-56.0)	34.0 (28.0-49.0)
Gender			
Male, No. (%)	254 (89.1)	166 (87.4)	88 (92.6)
Female, No. (%)	31 (10.9)	24 (12.6)	7 (7.4)
Route of transmission			
Homosexual transmission, No. (%)	170 (59.6)	109 (57.4)	61 (64.2)
Heterosexual transmission, No. (%)	91 (31.9)	63 (33.2)	28 (29.5)
Other, No. (%)	24 (8.4)	18 (9.5)	6 (6.3)
Body mass index, median (IQR), Kg/M^2^	21.6 (19.6-23.9)	21.5 (19.5-23.8)	22.2 (19.6-24.0)
<18.5 Kg/M^2^, No. (%)	37 (13.0)	23 (12.1)	14 (14.7)
18.5-23.9 Kg/M^2^, No. (%)	179 (62.8)	122 (64.2)	57 (60.0)
≥24 Kg/M^2^, No. (%)	69 (24.2)	45 (23.7)	24 (25.3)
HBV coinfection, No. (%)	33 (11.6)	18 (9.5)	15 (15.8)
HCV coinfection, No. (%)	14 (4.9)	10 (5.3)	4 (4.2)
Time between diagnosis and ART initiation, median (IQR), days	26.0 (13.0-68.0)	26.0 (13.0-61.0)	27.0 (12.0-348.0)
≤14 d, No. (%)	90 (31.6)	62 (32.6)	28 (29.5)
15-30 d, No. (%)	77 (27.0)	53 (27.9)	24 (25.3)
≥31 d, No. (%)	118 (41.4)	75 (39.5)	43 (45.3)
CD4+ T-cell count, median (IQR), cells/μL	84.0 (25.5-145.0)	103.0 (36.8-155.5)	56.0 (18.0-117.0)**
CD4 ≤50 cells/μL, No. (%)	100 (35.1)	56 (29.5)	44 (46.3)**
CD4/CD8 ratio, median (IQR)	0.12 (0.05-0.18)	0.13 (0.06-0.19)	0.09 (0.04-0.14)*
CD4/CD8 ratio ≤0.1, No. (%)	134 (47.0)	80 (42.1)	54 (56.8)*
Log_10_ VL, median (IQR), copies/mL	5.0 (4.6-5.4)	5.0 (4.6-5.3)	5.2 (4.8-5.6)**
VL ≥100,000 copies/mL, No. (%)	146 (51.2)	84 (44.2)	62 (65.3)**
Opportunistic infections, No. (%)	144 (50.5)	81 (42.6)	63 (66.3)***

IQR, Interquartile range; HBV, hepatitis B virus; HCV, hepatitis C virus; ART, antiretroviral therapy; VL, viral load; EFV, efavirenz; DTG, dolutegravir; *P<0.05; **P<0.01; ***P<0.001; this P-value for comparing the EFV and the DTG groups.

### Virological suppression

**Figure 2:**
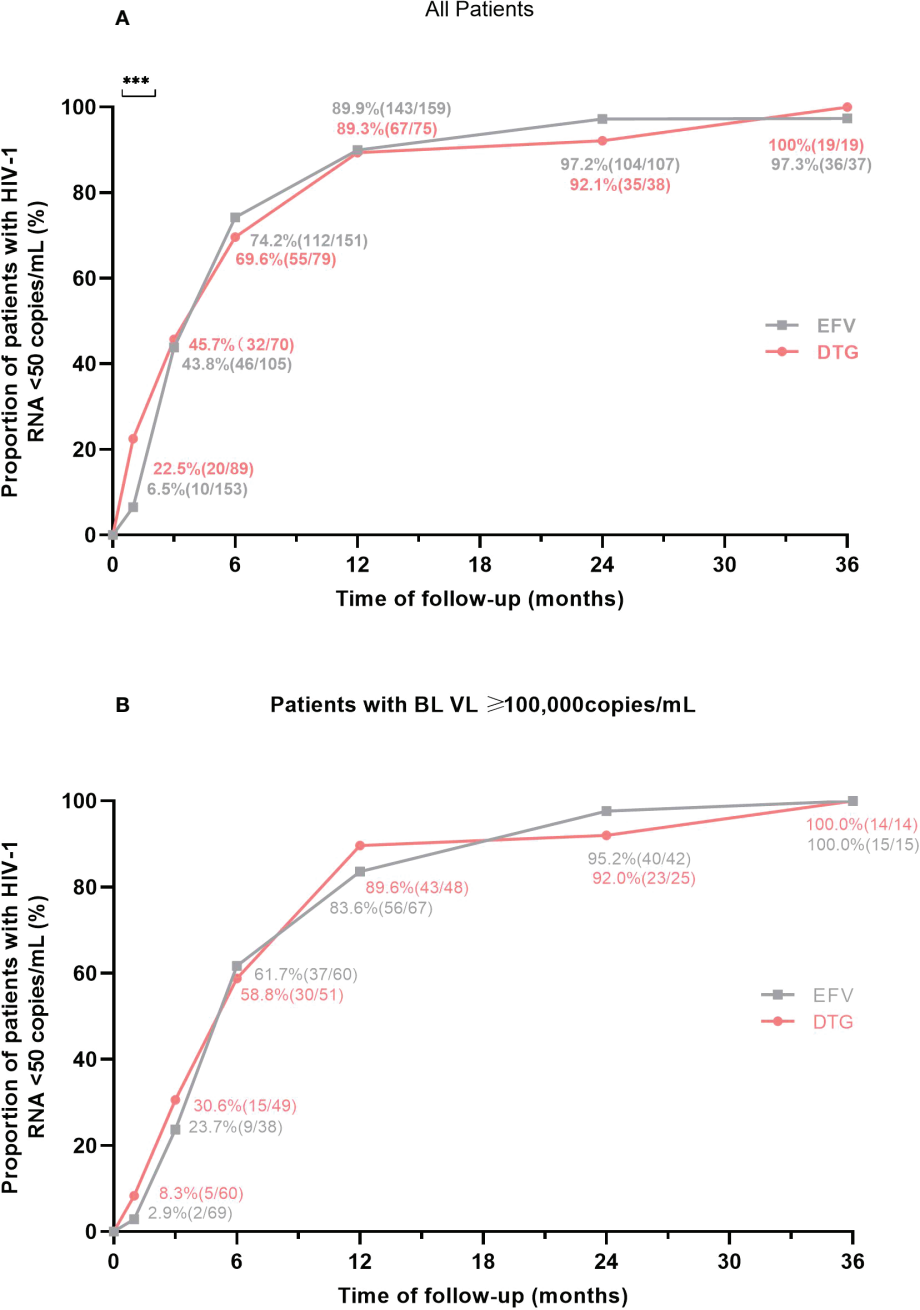
Proportion of patients with virological suppression at 6 different follow-up points according to antiretroviral regimens. **(A)** Proportion of virological suppression in all patients. **(B)** Proportion of virological suppression in patients with a baseline VL ≥ 100,000 copies/mL. BL, baseline; VL, viral load; EFV, efavirenz; DTG, dolutegravir. ***P<0.001.

The proportions of patients with virological suppression at 6 different follow-up points according to antiretroviral regimens were shown in **Figure 2**. After ART initiation, the proportion of patients with HIV-1 RNA < 50 copies/mL was higher (22.5% versus 6.5%, *P* < 0.001) in those on DTG-based regimens at month 1. Then, virological suppression rates in the DTG group were 45.7% (32/70) at month 3, 69.6% (55/79) at month 6, 89.3% (67/75) at month 12, 92.1% (35/38) at month 24, 100% (19/19) at month 36; and among those with EFV, the rates of virological suppression were 43.8% (46/105), 74.2% (112/151), 89.9% (143/159), 97.2% (104/107) and 97.3% (36/37) at month 3, 6, 12, 24 and 36, respectively (**Figure 2A**), which without intergroup significant difference at these follow-up points (all *P* > 0.05). In patients with a baseline VL ≥100,000 copies/mL, the proportion of two groups with HIV-1 RNA < 50 copies/mL were no significant difference (all *P* > 0.05) at 6 different follow-up points, including month 1, 3, 6, 12, 24 and 36 (**Figure 2B**). When patients were treated with low-dose EFV (400mg), virological suppression rate was also lower than the DTG group at month 1 (5.6% vs. 22.5%, *P*=0.025) and without significant difference at other points (**Supplementary Table 2**).

### Immunologic response

**Figure 3:**
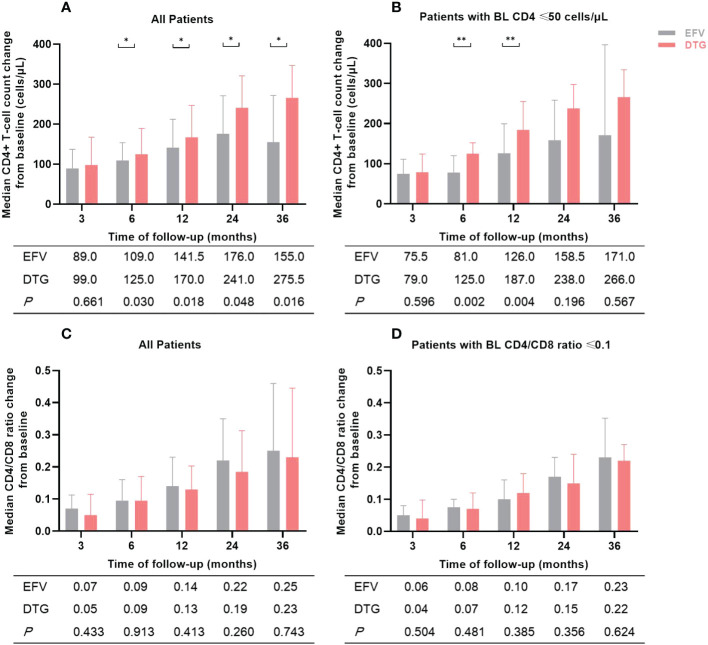
Changes in median CD4+ T-cell count and CD4/CD8 ratio of patients during the follow-up period according to antiretroviral regimens. **(A)** Median CD4+ T-cell count change from baseline in all patients. **(B)** Median CD4+ T-cell count change from baseline in patients with baseline CD4+ T-cell count ≤ 50 cells/μL. **(C)** Median CD4/CD8 ratio change from baseline in all patients. **(D)** Median CD4/CD8 ratio change from baseline in patients with baseline CD4/CD8 ratio ≤ 0.1. The numbers in the table below the figure were the median change of CD4+ T-cell count or CD4/CD8 ratio from baseline. BL, baseline; EFV, efavirenz; DTG, dolutegravir. *P<0.05; **P<0.01.

Changes in median CD4+ T-cell count and CD4/CD8 ratio of patients during the follow-up period were illustrated in **Figure 3**. Compared to baseline, median CD4+ T-cell count and CD4/CD8 ratio increased significantly at months 3, 6, 12, 24, and 36 after ART initiation both in the EFV and the DTG groups. However, the CD4+ T-cell count increased greater in patients on DTG-based regimens than in those with EFV after 6, 12, 24, and 36 months (all *P <*0.05) (**Figure 3A**). Furthermore, the DTG group had a greater increment of CD4+ T-cell count than the EFV group in patients with baseline CD4+ T-cell count ≤ 50 cells/μL at month 6 (*P*=0.002) and month 12 (*P*=0.004) (**Figure 3B**). The changes of CD4/CD8 ratio from baseline to 5 different follow-up points were similar between the two groups (all *P >*0.05, **Figures 3C, D**). When low-dose EFV (400mg) was compared with DTG, neither CD4+ T-cell count nor CD4/8 ratio was significantly different in the follow-up (**Supplementary Table 3**).

### Treatment discontinuation and IRIS

During the study period, a total of 52 (18.2%) patients discontinued EFV- or DTG-based regimens after ART initiation: 34 (65.4%) discontinuations occurred within 12 months and the other 18 (34.6%) after 12 months, and there was no difference between the two regimens in the rate of treatment discontinuation (**Table 2**). Within the first 12 months, there were 24 (12.6%) discontinued EFV, and the reasons for discontinuation were drug side effects (5, 20.8%), drug-drug interactions (3, 12.5%), absence of viral suppression (1, 4.2%), patient request for other regimens (1, 4.2%), convenience (1, 4.2%), ART discontinuation by patients (6, 25.0%), loss of follow-up (2, 8.3%), and death (5, 20.8%); while the reasons were drug side effects (3, 30.0%), absence of viral suppression (1, 10.0%), patient request for other regimens (1, 10.0%), and death (5, 50.0%) in 10 (10.5%) DTG discontinuers. After 12 months, the main reasons for treatment discontinuations were drug side effects (5, 45.5%), low adherence and resistance to EFV (3, 27.3%) in the EFV group and patients request for other regimens (3, 42.9%), drug side effects (2, 28.6%) and convenience (2, 28.6%) in the DTG group. Of note, 11 patients died during follow-up. The common causes of death included progressive multifocal leukoencephalopathy (27.3%, 3/11), cancer (27.3%, 3/11), malignant lymphoma (18.2%, 2/11) (**Supplementary Table 4**).

**Table 2:** Treatment discontinuation during the follow-up period.

	Before 12 months			After 12months		
	EFV	DTG	*P*	EFV	DTG	*P*
Regimen discontinuation, No. (%)>	24 (12.6)	10 (10.5)	0.605	11 (6.6)	7 (8.2)	0.640
Reasons, No. (%)						
Drug side effect	5 (20.8)	3 (30.0)		5 (45.5)	2 (28.6)	
Drug-drug interactions	3 (12.5)	0		1 (9.1)	0	
Absence of viral suppression	1 (4.2)	1 (10.0)		0	0	
Low adherence and resistance	0	0		3 (27.3)	0	
Patients request for other regimens	1 (4.2)	1 (10.0)		1 (9.1)	3 (42.9)	
Convenience	1 (4.2)	0		0	2 (28.6)	
ART discontinuation by patients	6 (25.0)	0		0	0	
Loss of follow-up	2 (8.3)	0		0		
Death	5 (20.8)	5 (50.0)		1 (9.1)	0	

ART, antiretroviral therapy; EFV, efavirenz; DTG, dolutegravir.

The incidence of IRIS was very low in our study, with only 7 cases reported overall (4/95 with DTG-, and 3/190 with EFV-based regimens) and without significant difference between ART regimens (*P*=0.224). Among PLWH on DTG-based 3DRs, 2 IRIS events were related to pulmonary tuberculosis, to pneumocystis pneumonia in 1 patient, and to cryptococcal meningitis in 1 patient. Among those on EFV-based 3DRs, 3 IRIS events were related to cryptococcal meningitis, talaromyces marneffei infection or pulmonary tuberculosis, and lymphatic tuberculosis, respectively. Patients with IRIS events maintained their original anti-infective regimen, with additional anti-inflammatory therapy in 6 patients and no specific anti-inflammatory therapy in 1 patient whose IRIS event related to pneumocystis pneumonia. All 7 PLWH were discharged in improved condition, and none have died to date.

### Factors of immune recovery

**Figure 4:**
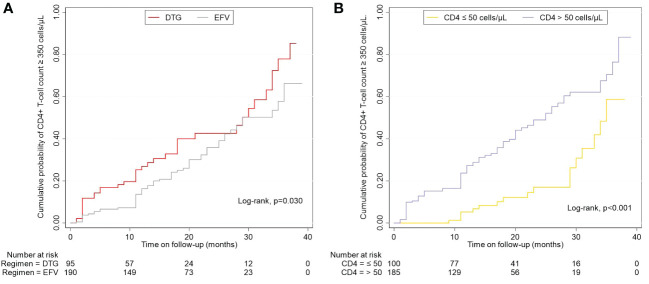
Effects of regimens and baseline CD4+ T-cell count on the cumulative probability of CD4+ T-cell count ≥ 350 cells/μL. **(A)** Different regimens on CD4+ T-cell count ≥ 350 cells/μL. **(B)** Baseline CD4+ T-cell count-stratified results on CD4+ T-cell count ≥ 350 cells/μL. EFV, efavirenz; DTG, dolutegravir.

Overall, 34.0% (97/285) achieved a CD4+ T-cell count ≥ 350 cells/μL during follow-up, and the median time taken to achieve immune recovery was 29.0 months (IQR 24.2-33.8). **Figure 4A** showed that patients treated with DTG outperformed patients with EFV in the rate of immune recovery (log-rank test, *P*=0.030), despite the median time to achieve a CD4+ T-cell count ≥ 350 cells/μL was 29 months in both DTG and EFV groups. As shown in **Figure 4B**, patients with baseline CD4+ T-cell count > 50 cells/μL were superior to those with baseline CD4+ T-cell count ≤ 50 cells/μL in the probability of immune recovery (log-rank test, *P* < 0.001), and the median time to immune recovery were 25 month and 35 months, separately. Univariate Cox regression models analysis showed that the immune recovery was associated with age (per 10 years increased, hazard ratio, HR = 0.727, 95% confidence interval, CI: 0.618-0.854, *P* < 0.001), baseline CD4+ T-cell count (per 50 cells/μL increase, HR = 2.860, 95% CI: 1.682-4.863, *P* < 0.001), baseline CD4/CD8 ratio (per 0.1 increase, HR = 1.327, 95% CI: 1.129-1.559, *P* = 0.001), baseline VL (per 1 Log10 increase, HR = 1.384, 95% CI: 1.028-1.862, *P* = 0.032) and DTG-based regimens (HR = 1.557, 95% CI: 1.033-2.347, *P* = 0.034) compared to regimens contained EFV. In the multivariate analysis, the factors of age (per 10 years increased, HR = 0.743, 95% CI: 0.629-0.877, *P* < 0.001), baseline CD4+ T-cell count (per 50 cells/μL increase, HR = 3.030, 95% CI: 1.800-5.099, *P* < 0.001), baseline VL (per 1 Log_10_ increase, HR = 1.895, 95% CI: 1.380-2.603, *P* = <0.001) and DTG (HR = 1.696, 95% CI: 1.089-2.642, *P* = 0.019) compared to EFV were associated with the CD4+ T-cell count ≥ 350 cells/μL after adjusting for potential confounders (**Table 3**). About the CD4/CD8 ratio, only 2.1% (6/285) achieved a CD4/CD8 ratio ≥ 1 overall during follow-up.

**Table 3:** Cox regression analyses for immune recovery.

	Univariate analysis		Multivariate analysis	
	HR (95% CI)	*P*	HR (95% CI)	*P*
Age, per 10 years increased	0.727 (0.618-0.854)	<0.001	0.743 (0.629-0.877)	<0.001
Gender				
Men	1.087 (0.579-2.039)	0.795		
Female	Reference			
Body mass index, Kg/M^2^				
18.5-23.9	Reference			
<18.5	1.140 (0.646-2.011)	0.651		
≥24	1.044 (0.642-1.698)	0.861		
Route of transmission				
Homosexual transmission	Reference			
Heterosexual transmission	0.852 (0.549-1.324)	0.477		
HBV coinfection	1.042 (0.580-1.874)	0.890		
HCV coinfection	0.454 (0.112-1.845)	0.270		
CD4+T-cell count, per 50 cells/μL increase	2.860 (1.682-4.863)	<0.001	3.030 (1.800-5.099)	<0.001
CD4+ T-cell count*LN (T_)	0.776 (0.636-0.947)	0.013		
CD4/CD8 ratio, per 0.1 increase	1.327 (1.129-1.559)	0.001		
Log_10_ VL, per 1 Log_10_ increase	1.384 (1.028-1.862)	0.032	1.895 (1.380-2.603)	<0.001
Time between diagnosis and initiation of ART, days				
≤14	Reference			
15-30	0.706 (0.423-1.178)	0.183		
≥31	0.740 (0.464-1.180)	0.206		
Opportunistic infections exist	0.859 (0.576-1.283)	0.458		
Treatment regimens				
EFV-based	Reference			
DTG-based	1.557 (1.033-2.347)	0.034	1.696 (1.089-2.642)	0.019

HR, hazard ratio; 95% CI, 95% confidence interval; HBV, hepatitis B virus; HCV, hepatitis C virus; VL, viral load; ART, antiretroviral therapy; EFV, efavirenz; DTG, dolutegravir.

## Discussion

The proportion of advanced ART-naïve PLWH reached 32.04% (406/1267) between March 1, 2019, and May 31, 2021, at The Second Hospital of Nanjing, China. There were many available ART regimens among advanced ART-naïve PLWH, and EFV- and DTG-based 3DRs were more common options. In our study, DTG showed a faster decline in VL and a faster increase in absolute CD4+ T-cell count in respect of EFV. However, no statistical differences were observed in the increase in CD4/CD8 ratio and safety between EFV- and DTG-based regimens. In addition to different ART regimens, age, VL, and CD4+ T-cell count before ART initiation were also associated with immune recovery.

Previous studies (3, 5, 12) have shown that the proportion of advanced ART initiation was above 20% regardless of the level of local economic development, and the proportion was even 32% in this study, the ART regimens choice and efficacy of this group of PLWH should not be ignored. Integrase strand transfer inhibitor (INSTI) had been reported the better efficacy and safety than non-nucleoside reverse transcriptase inhibitor (NNRTI) and protease inhibitors (PI) (23–25), and DTG was an effective and accessible second-INSTI with low risk of resistance (26). A pooled study found that DTG had better viral suppression at 4 weeks after initiation of ART than EFV (OR: 9.81; 95% CI: 7.83-12.25) (27). In this study, the proportions of plasma virological suppression were similar between the two groups at month 3, 6, 12, 24, and 36, but the DTG group showed a significantly higher proportion than the EFV group at month 1 (*P* < 0.05), regardless of the dose of EFV. The foundation supported the notion that rapid reduction of plasma VL with DTG-based regimens in PLWH (28) was equally applicable in patients with advanced HIV infection. What’s more, rapid virological suppression may be associated with immune recovery, and reduce the rate of HIV transmission in the population. There was no difference in early virological suppression between the two groups in patients with a baseline VL > 10^5^ copies/mL in our study, which was consistent with the results of phase III clinical trial of DTG (29), but this was also only a phenomenon and the exact clinical significance was not yet clear.

All ART regimens can promote immune recovery in PLWH with sustained viral suppression in long-term ART, but there is still no clear conclusion as to whether ART drug classes and regimens lead to different immune recovery (30). Some studies reported that ART regimens containing INSTI were associated with greater increases in CD4+ T-cell count compared to the NNRTI (23, 27, 31, 32) while others showed no difference (33, 34). In this follow-up study, although CD4+ T-cell count increased significantly both in the DTG and the EFV groups, the increase was greater in the DTG group than in the EFV group. Moreover, DTG-based regimens still showed a more rapid increase in CD4+ T-cell count when the study population contained patients with severe immunodeficiency with CD4 ≤ 50 cells/μL. In a multicenter study conducted in Italy (9), advanced PLWH in the DTG group had faster CD4+ T-cell counts than patients without DTG, and our results were consistent with it. However, if only 400mg EFV and DTG were compared, the change of CD4+ T-cell count was no significant difference in this study. We thought severe and complex clinical conditions of patients in the DTG group and small number of patients in the low-dose EFV group may have affected our results. In addition to CD4+ T-cell count, CD4/CD8 ratio is also an important indicator of immune recovery, which can predict non-AIDS-event morbidity and mortality independently of CD4+ T-cell count (35–37). So the CD4/CD8 ratio was included in this study to compare the difference between DTG and EFV on immune recovery. Most studies revealed that INSTI had a greater CD4/CD8 ratio increase than NNRTI and PI (38, 39), but in the *post hoc* analysis of the SINGLE trial (40), the EFV group had a higher proportion of PLWH with CD4/CD8 ratio ≥ 1 than DTG at week 96. However, similar changes of the CD4/CD8 ratio had been reported for different ART regimens in acutely/recently infected PLWH (41). Our study found that there was no difference between DTG- and EFV-based regimens on CD4/CD8 ratio gains. Considering that the DTG group had a lower CD4/CD8 ratio at baseline, which may affect the change of CD4/CD8 ratio during follow-up, we also analyzed patients with baseline CD4/CD8 ≤ 0.1 separately. The results still showed a non-significant difference in CD4/CD8 ratio change between the two groups, probably because the DTG group had a rapid increase in CD4 cells along with a faster increase in CD8 cells compared to the EFV group.

The literature on the choice of treatment options for ART-naïve patients with advanced HIV infection is rare, especially in China. Generally, physicians consider the convenience of ART regimens, patient conditions, and treatment experience when choosing a regimen for advanced ART-naïve PLWH (42). Although the reasons for the treatment options for the patients in this study were not precisely available, it could be speculated from the baseline characteristics of both groups that the baseline conditions of the patients pre-ART initiation affected the choice of ART regimens, as reflected by the fact that higher proportion of patients with low CD4 and CD4/CD8 and high VL were in the DTG group. Moreover, it was evident by the reasons for treatment discontinuation that the drug side effects, the patients’ ideas, and the convenience of the drug also influenced the treatment options in this study. However, we did not observe the difference between DTG and EFV in treatment discontinuation, which was similar to another study (9) that included patients initiating DTG or non-DTG with CD4 < 350 cells/μL or with AIDS-defining events. A total of 30 patients who were resistant or allergic to EFV and chose EFV were excluded from our study, while no patients in the DTG group excluded because of the same criteria. On the one hand, we did not test baseline drug resistance for DTG, but none of them discontinued DTG due to resistance, this phenomenon indicated that DTG had a higher barrier to resistance than EFV for most PLWH. On the other hand, there were still a small number of patients in clinical practice that chose EFV-based regimens that were not suitable for them due to their conditions. And this study could not provide a treatment reference for these patients, but pre-ART drug resistance should be carefully considered when EFV-based regimens used in China. It was noteworthy that four patients discontinued regimens due to drug-drug interaction in the EFV group, while none were in the DTG group in our study. The higher incidence of co-infections but fewer drug-drug interactions in the DTG group suggested that we can prioritize DTG over EFV when patients have other treatments in parallel. In total, 11 (3.9%) patients died during the follow-up and the vast majority of deaths (90.9%, 10/11) occurred within 1 year. The causes of death were progressive multifocal leukoencephalopathy, cancer, malignant lymphoma, AIDS-associated cachexia, and cardiovascular diseases. This study only included immunocompromised advanced PLWH, which was more susceptible to opportunistic infections or tumors that lead to their death. In clinical practice, once a patient is diagnosed as HIV-1 positive, he or she should be encouraged to initiate ART as soon as possible, and to test VL and immune function indicators regularly for monitoring treatment efficacy. Especially for advanced PLWH with poor immune function, more frequent lymphocyte testing and visits should be given, and education on medication adherence should be reinforced, along with medications to prevent infection, such as cotrimoxazole.

In addition to treatment discontinuation, we used IRIS as an indicator to evaluate the safety, which associated with rapid VL decay and rapid immune restoration (43, 44). One study showed the incidence of IRIS was nearly twice as high in the INSTI group as in the non-INSTI group (45), and Psichogiou et al. found that INSTIs were independent risk factors for the emergence of IRIS compared to NNRTIs (OR 2.89; 95% CI 1.26-6.64; *p* = 0.012) (46). However, in the present study, despite rapid viral suppression and greater CD4+ T-cell count increase in the DTG group, the diagnosis of IRIS did not higher than the EFV group (*P*=0.224). Our results were similar to those of a South Korean study conducted in patients with CD4+ T-cell count < 200 cells/μL, which also had IRIS mostly related to tuberculosis (47).

The study also analyzed the factors influencing immune recovery in patients with advanced HIV infection. The results indicated the association between ART regimens and immune recovery remained independent; DTG- regimens were still more likely to achieve immune recovery than EFV-based regimens. Other indicators of significance included age, baseline plasma viral load, and CD4+ T-cell count. As previously reported (48, 49), we found that the older were more difficult to achieve immune recovery, possibly because the physiological degeneration of the thymus limited their ability to produce naïve T cells (50, 51). Pre-ART VL and CD4+ T-cell count ≥ 350 cells/μL were positively correlated as reported in other research (52–54). This correlation can be explained by the number of CD4 T cells sequestered in lymphoid tissue may be higher in the presence of uncontrolled viral replication, and can be rapidly released into the peripheral circulation after ART initiation (55, 56). Higher CD4+ T-cell count was associated with better immune recovery, suggesting that recovery of CD4+ T-cell count was at least partially negatively correlated with the severity of CD4+ T cell depletion. A prospective study suggested that HIV-HCV coinfection might reduce the growth rate of CD4+ T-cell count (57). However, we did not find an independent effect of HCV infection on immune recovery in patients with advanced HIV infection. This might be related to the small number of patients with combined HCV in the study. Rapid initiation of ART after HIV diagnosis was beneficial for improving virological suppression, accelerating immune recovery, and reducing morbidity and mortality rates (58–61). In this study, 31.6% of PLWH initiated ART within 14 days of diagnosis, but there was no significant difference in immune recovery compared to PLWH who initiated ART after 14 days. On the one hand, advanced ART-naïve patients had very low CD4+ T-cell count and CD4/8 ratio, and severely impaired immune function limited its recovery. On the other hand, opportunistic infections might lead to delayed ART (62, 63), which affected immune recovery. It’s more likely to achieve immune reconstitution in all PLWH without opportunistic infections (64). In contrast, this study was conducted in patients with advanced HIV infection, where the incidence of opportunistic infections was nearly half, and the association between opportunistic infections and immune reconstitution may have been obscured. Studies on the factors associated with immune recovery in advanced ART-naïve patients are rare, and the mechanisms and factors influencing immune recovery in this group of patients with severe immune impairment may need further exploration.

Our study has some limitations. First, it is a retrospective non-randomized study; there may be missing data on patients at different follow-up time points despite the availability of well-preserved follow-up records of PLWH at The Second Hospital of Nanjing. In addition, the study is a single-center design and relatively small sample size, which may not be representative of the national population; a multicenter study with a large sample is needed for further study. Third, the characteristics of the two groups were not perfectly balanced at baseline, which could be attributed to the clinical situations of the patients and the different prescribing attitudes of the physicians in their regular clinical practice. Lastly, the convenience of the drug might affect patients’ choice, but single-tablet drugs such as bictegravir/emtricitabine/TAF (BIC/FTC/TAF) were used in small numbers during the study period, and thus not included in the study analysis. Further attention to drug selection and efficacy differences in patients with advanced HIV infection is needed because of changes in health insurance policies, significant price reductions for some single-tablet drugs, and more drug choices for patients.

## Conclusion

In this retrospective study, EFV- and DTG-based 3DRs were more common options in ART-naïve patients with advanced HIV infection, and it appeared that DTG-based regimens were better options for initial therapy compared to regimens including EFV. In addition to ART regimens, age, baseline plasma VL and CD4+ T-cell count were also associated with immune recovery among advanced ART-naïve PLWH.

## Data availability statement

The raw data supporting the conclusions of this article will be made available by the authors, without undue reservation.

## Ethics statement

The studies involving human participants were reviewed and approved by the Ethics Committee of the Second Hospital of Nanjing (Nanjing, China). The patients/participants provided their written informed consent to participate in this study.

## Author contributions

HW, ChC and MZ conceived and designed the study, and H Wei led at study implementation. MZ, ML, MQ, YS, NY, RL, ZY, XZ, XX, and CC collected the study data. MZ and ML analyzed the data and wrote the draft manuscript. All authors commented on previous versions of the manuscript. ChC and HW revised the manuscript. All authors contributed to the article and approved the submitted version.
