# Supplementary material for: A retrospective clinical study of dolutegravir- versus efavirenz-based regimen in treatment-naïve patients with advanced HIV infection in Nanjing, China
Source: Front Immunol. 2023 Jan 9;13:1033098. doi: 10.3389/fimmu.2022.1033098 (PMC9868135; doi:10.3389/fimmu.2022.1033098)
Supplement: Supplementary file 1 [file DataSheet_1.pdf]

Supplementary Table 1 Baseline characteristics between EFV (400mg) and DTG

	400mg EFV (n=46)	DTG (n=95)	*P*
Age, median (IQR), y	42.0 (27.8-56.3)	34.0 (28.0-49.0)	0.254
Gender			**0.032**
Male, No. (%)	37 (80.4)	88 (92.6)	
Female, No. (%)	9 (19.6)	7 (7.4)	
Route of transmission			0.137*
Homosexual transmission, No. (%)	24 (52.2)	61 (64.2)	
Heterosexual transmission, No. (%)	21 (45.7)	28 (29.5)	
Other, No. (%)	1 (2.2)	6 (6.3)	0.137*
Body mass index, median (IQR), Kg/M^2	20.3 (19.0-22.3)	22.2 (19.6-24.0)	**0.016**
<18.5 Kg/M^2, No. (%)	6 (13.0)	14 (14.7)	0.110
18.5-23.9 Kg/M^2, No. (%)	35 (76.1)	57 (60.0)	
≥24 Kg/M^2, No. (%)	5 (10.9)	24 (25.3)	
HBV coinfection, No. (%)	3 (6.5)	15 (15.8)	0.122
HCV coinfection, No. (%)	2 (4.3)	4 (4.2)	>0.999*
Time between diagnosis and ART initiation, median (IQR), days	26.0 (12.8-84.5)	27.0 (12.0-348.0)	0.697
≤14 d, No. (%)	13 (28.3)	28 (29.5)	0.931
15-30 d, No. (%)	13 (28.3)	24 (25.3)	
≥31d, No. (%)	20 (43.5)	43 (45.3)	
CD4+ T-cell count, median (IQR), cells/μL	112.0 (56.8-162.5)	56.0 (18.0-117.0)	**0.002**
CD4 ≤50 cells/μL, No. (%)	10 (21.7)	44 (46.3)	**0.005**
CD4/CD8 ratio, median (IQR)	0.17 (0.09-0.23)	0.09 (0.04-0.14)	**<0.001**
CD4/CD8 ratio ≤0.1, No. (%)	12 (26.1)	54 (56.8)	**0.001**
Log_{10} VL, median (IQR), copies/mL	4.9 (4.5-5.4)	5.2 (4.8-5.6)	0.060
VL ≥100,000 copies/mL, No. (%)	20 (43.5)	62 (65.3)	**0.014**
Opportunistic infections, No. (%)	16 (34.8)	63 (66.3)	**<0.001**

IQR, Interquartile range; HBV, hepatitis B virus; HCV, hepatitis C virus; ART, antiretroviral therapy; VL, viral load; EFV, efavirenz; DTG, dolutegravir; **P* Fisher's exact test.

Supplementary Table 2 Differences in virological suppression rates between EFV (400 mg) and DTG at different follow-up points

	400mg EFV (n=46)	DTG (n=95)	*P*
1 month	5.6% (2/36)	22.5% (20/89)	**0.025**
3 month	56.0% (14/25)	45.7% (32/70)	0.377
6 month	71.4% (30/42)	69.6% (55/79)	0.836
12 month	95.5% (42/44)	89.3% (67/75)	0.320*
24 month	96.2% (25/26)	92.1% (35/38)	0.640*
36 month	100% (5/5)	100% (19/19)	>0.999*

EFV, efavirenz; DTG, dolutegravir. **P* Fisher's exact test.

Supplementary Table 3 Changes in median CD4+ T-cell count and CD4/CD8 ratio of patients (400mg EFV vs. DTG) during the follow-up period

	400mg EFV (n=46)	DTG (n=95)	*P*
CD4+ T-cell count (cells/μL)			
3 month (27 vs. 82)	123.0 (61.0-190.0)	99.0 (42.8-168.0)	0.599
6 month (39 vs.79)	121.0 (74.0-147.0)	125.0 (82.0-192.0)	0.306
12 month (43 vs. 75)	136.0 (87.0-220.0)	170.0 (116.0-251.0)	0.113
24 month (26 vs. 39)	218.0 (136.0-299.5)	241.0 (149.0-338.0)	0.560
36 month (5 vs. 18)	99.0 (91.0-194.5)	275.5 (187.0-342.5)	0.007
CD4/CD8 ratio			
3 month (27 vs. 81)	0.08 (0.04-0.16)	0.05 (0.03-0.12)	0.376
6 month (39 vs. 78)	0.10 (0.06-0.17)	0.09 (0.05-0.17)	0.819
12 month (43 vs. 74)	0.16 (0.10-0.26)	0.13 (0.09-0.20)	0.194
24 month (26 vs. 38)	0.29 (0.14-0.38)	0.19 (0.12-0.31)	0.151
36 month (5 vs. 17)	0.23 (0.15-0.43)	0.23 (0.15-0.45)	0.784

The number in the table was the median change of CD4+ T-cell count or CD4/CD8 ratio from baseline. EFV, efavirenz; DTG, dolutegravir.

Supplementary Table 4 Information of 11 patients who died during the follow-up period

Number	VL at baseline (copies/mL)	CD4 cells count at baseline (cells/μL)	ART start date	ART regimens	Date of death	Last VL before death (copies/mL)	Last CD4 cells count before death (cells/μL)	Cause of death
1	388000	80	2019/5/8	EFV+TDF+3TC	2019/11/4	588000	No data	AIDS-associated cachexia
2	28800	196	2019/9/18	EFV+TDF+3TC	2019/12/18	<20	90	Stomach cancer
3	55700	167	2019/5/22	EFV+TDF+3TC	2020/1/13	<20	180	Malignant lymphoma
4	256000	20	2019/3/20	EFV+TDF+3TC	2020/1/14	<20	68	Lung cancer
5	420000	11	2020/9/25	EFV+TDF+3TC	2020/11/17	No data	No data	Unable to determine
6	292000	44	2020/8/27	DTG+TDF+3TC	2020/11/17	150	155	Progressive multifocal leukoencephalopathy
7	240000	61	2020/6/28	DTG+TDF+3TC	2020/12/31	101	59	Progressive multifocal leukoencephalopathy
8	20200	52	2020/12/22	DTG+TDF+3TC	2021/3/9	25.3	70	Progressive multifocal leukoencephalopathy
9	169000	49	2021/4/14	DTG+TDF+3TC	2021/8/14	70.7	14	Malignant lymphoma
10	6830	198	2020/9/9	EFV+TDF+3TC	2021/11/4	<20	87	Esophageal cancer
11	120000	5	2021/1/27	DTG+TDF+3TC	2021/12/26	76.2	69	Cardiovascular diseases

VL, viral load; ART, antiretroviral therapy; EFV, efavirenz; TDF, tenofovir disoproxil fumarate; 3TC, lamivudine; DTG, dolutegravir.
